# Digital Self-Management Platform for Adult Asthma: Randomized Attention-Placebo Controlled Trial

**DOI:** 10.2196/50855

**Published:** 2024-04-29

**Authors:** Aaron Kandola, Kyra Edwards, Joris Straatman, Bettina Dührkoop, Bettina Hein, Joseph Hayes

**Affiliations:** 1 Medical Research Council Unit of Lifelong Health and Aging University College London London United Kingdom; 2 juli Health Hull, MA United States; 3 Division of Psychiatry University College London London United Kingdom; 4 Camden and Islington NHS Foundation Trust London United Kingdom

**Keywords:** asthma, mobile health, self-management, randomized controlled trial, randomized, controlled trial, controlled trials, RCT, RCTs, respiratory, pulmonary, smartphone, platform, digital health, chronic, breathing, disease management, mHealth, mobile health, app, apps, application, applications, mobile phone

## Abstract

**Background:**

Asthma is one of the most common chronic conditions worldwide, with a substantial individual and health care burden. Digital apps hold promise as a highly accessible, low-cost method of enhancing self-management in asthma, which is critical to effective asthma control.

**Objective:**

We conducted a fully remote randomized controlled trial (RCT) to assess the efficacy of juli, a commercially available smartphone self-management platform for asthma.

**Methods:**

We conducted a pragmatic single-blind, RCT of juli for asthma management. Our study included participants aged 18 years and older who self-identified as having asthma and had an Asthma Control Test (ACT) score of 19 or lower (indicating uncontrolled asthma) at the beginning of the trial. Participants were randomized (1:1 ratio) to receive juli for 8 weeks or a limited attention-placebo control version of the app. The primary outcome measure was the difference in ACT scores after 8 weeks. Secondary outcomes included remission (ACT score greater than 19), minimal clinically important difference (an improvement of 3 or more points on the ACT), worsening of asthma, and health-related quality of life. The primary analysis included participants using the app for 8 weeks (per-protocol analysis), and the secondary analysis used a modified intention-to-treat (ITT) analysis.

**Results:**

We randomized 411 participants between May 2021 and April 2023: a total of 152 (37%) participants engaged with the app for 8 weeks and were included in the per-protocol analysis, and 262 (63.7%) participants completed the week-2 outcome assessment and were included in the modified ITT analysis. Total attrition between baseline and week 8 was 259 (63%) individuals. In the per-protocol analysis, the intervention group had a higher mean ACT score (17.93, SD 4.72) than the control group (16.24, SD 5.78) by week 8 (baseline adjusted coefficient 1.91, 95% CI 0.31-3.51; *P*=.02). Participants using juli had greater odds of achieving or exceeding the minimal clinically important difference at 8 weeks (adjusted odds ratio 2.38, 95% CI 1.20-4.70; *P*=.01). There were no between group differences in the other secondary outcomes at 8 weeks. The results from the modified ITT analyses were similar.

**Conclusions:**

Users of juli had improved asthma symptom control over 8 weeks compared with users of a version of the app with limited functionality. These findings suggest that juli is an effective digital self-management platform that could augment existing care pathways for asthma. The retention of patients in RCTs and real-world use of digital health care apps is a major challenge.

**Trial Registration:**

International Standard Randomised Controlled Trial Number (ISRCTN) registry ISRCTN87679686; https://www.isrctn.com/ISRCTN87679686

## Introduction

Asthma is one of the most common chronic conditions worldwide, with an increasing prevalence that currently affects 1 in 10 people at some time [1-3]. The inflammatory disease causes mild-to-severe respiratory symptoms, including shortness of breath, chest tightness, wheezing, and cough. It significantly burdens patients and health care services, including the need for long-term treatment, emergency care, and hospitalizations that will cost the US economy an estimated US $300 billion over the next 20 years in direct health care expenditure [4]. Effective asthma control is necessary to reduce these costs and improve the quality of life for people with the condition.

Asthma management is based on achieving symptom control and reducing the frequency and severity of exacerbations [5]. This involves the use of inhaled anti-inflammatory medications and the avoidance of asthma triggers. Symptom control is associated with improved quality of life, reduced health care costs, and better work performance [6]. However, a significant proportion of individuals with asthma have suboptimal control because of poor adherence to medication, insufficient recognition of triggers, comorbidities (such as rhinitis or obesity), health behaviors (such as smoking), and inadequate information about treatment [7]. Mobile apps may address some of these treatment challenges by enabling people with asthma to more easily and consistently self-manage their condition compared to existing treatment plans. For example, digital apps can offer timely reminders to improve medication adherence or real-time feedback to identify and adapt to possible triggers and health behaviors [8,9].

A 2017 systematic review and meta-analysis of randomized controlled trials (RCTs) of mobile, web-based, and messaging service apps to support asthma self-management [9] concluded that these interventions could improve asthma control, but that effectiveness and important features of the apps varied. The majority of these apps included combinations of medication prompts, patient education, digital diaries, action plans, and professional support facilitation [9]. A similar 2018 review of RCTs and observational studies concluded that, in adults with asthma, mobile apps were more effective than other types of digital interventions, such as web-based interventions [10]. Studies of app-based interventions published since these reviews have generally been feasibility trials or small underpowered RCTs [11-14]. A 2022 Cochrane review examined the effect of digital apps on asthma medication adherence, concluding they were likely to be useful in poorly adherent populations, but again highlighting heterogeneity among mobile or web-based interventions [8]. Despite the mixed evidence for effectiveness, several apps are publicly available. These apps frequently incorporate behavior change techniques and gamification. Reviews of these apps have highlighted that they vary considerably in quality, use a range of behavior change techniques, struggle with adequate engagement and retention, and lack clinical validation of efficacy [15-17]. The *Global Strategy for Asthma Management and Prevention* (Global Initiative for Asthma [GINA]) highlights that, despite the use of digital technologies rapidly increasing in patients with asthma, “high-quality studies are needed to evaluate their utility and effectiveness” [3].

We aimed to address the fundamental issue that commercially available apps require sufficient evaluation of their effectiveness, by conducting an RCT of juli. This is a digital health app that aims to support people with asthma by combining numerous approaches that have been shown as effective in research-grade apps for asthma, including symptom tracking; medication reminders; trigger identification (including geolocated weather, pollen, and air pollution data); data visualization of respiratory symptoms, mood, exercise, activity, sleep, and heart rate variability; and behavioral activation recommendations about how to improve these parameters [18,19]. Our RCT was fully remote, increasing time efficiency, cost-effectiveness, and reach. We hypothesized that participants randomized to juli would have a greater reduction in asthma symptoms at 8 weeks than those randomized to the attention-placebo control.

## Methods

### Study Design and Participants

We conducted a fully remote pragmatic single-blind, placebo-control RCT to test the efficacy of juli in adults with asthma. The trial was open to individuals from anywhere in the world, provided they were aged 18 to 65 years, English-speaking, had access to a smartphone, and self-identified as having asthma. We also only included people with asthma symptoms that were uncontrolled according to a score of 19 or lower on the Asthma Control Test (ACT) at baseline. An ACT score of 19 is consistent with GINA-defined uncontrolled asthma [20].

### Recruitment

Recruitment ran from May 2021 until April 2023. We recruited via self-help groups for asthma, online adverts, and social media posts. For the duration of the RCT, we modified the onboarding so that recruitment was automated with study information provided to participants within the app. The support for potential participants interested in the RCT was provided by this study’s team via email.

### Ethical Considerations

The University College London Ethics Committee gave full ethical approval (19413/001). All participants supplied written informed consent within the app, with additional information on a dedicated web page. Data required for the RCT were stored separately in an anonymized format. The juli app is Health Insurance Portability and Accountability Act, Service Organization Control Type 2, and General Data Protection Regulation compliant. Participants in both arms of the RCT were entered into a prize draw at 2, 4, 6, and 8 weeks with the possibility of winning US $20 at each time point. The trial was entered on the International Standard Randomised Controlled Trial Number (ISRCTN) registry (ISRCTN87679686). At the same time, we were running an RCT of the juli app for depression. This RCT had a similar design and analysis [21].

### Randomization and Masking

We assigned participants in a 1:1 ratio to either an attention-placebo control or the full version of juli. We automated and conducted randomization within the app, using random block sizes ranging from 4 to 8. To ensure data integrity, the treatment allocation was concealed from both the research team and independent statisticians until the analysis was finalized.

### Intervention

The juli app was developed by gamification experts in collaboration with patients, a psychiatrist, and a pulmonologist. A patient with asthma and a psychiatrist with expertise in mental health and physical health interface are the chief technical officer and chief medical officer of juli, respectively. We held development and user testing interviews with 10 patients with asthma (5 female individuals, aged 18-65 years). The app underwent multiple iterations following feedback from these patient panel interviews and discussions with a pulmonologist. Our trial used a full version of the juli app for the intervention group and a limited version in the attention-placebo control group. Participants with the complete juli app received automatic prompts to open the app each day at a user-inputted time. The app asked participants about how their asthma was affecting them on a 5-face emoji scale, their emergency inhaler use that day, how often they had a shortness of breath episode, and whether they woke in the night due to shortness of breath. Individuals could also track various factors they regarded as relevant to their asthma symptoms, such as tobacco smoke exposure [19]. The app connects to smart peak flow meters (such as Smart Peak Flow [Smart Respiratory Products Ltd] or MIR Smart One [Smart One]) through Google Fit or Apple HealthKit, or participants could enter this information manually.

The app presented participants with regular, geolocated weather, pollen, and air pollution data relevant to their asthma [22]. All participants could also access passively gathered smartphone data on relevant health-related factors, including, activity, menstrual cycle, and sleep. Participants could check this information daily and see associations with their asthma [23-25]. If they had them, participants with wearables that they chose to connect to the app would see additional data on workouts and heart rate variability, as well as improved data on activity and sleep. However, the lack of access to a wearable was not an exclusion criterion.

The app also uses behavioral activation techniques to provide personalized recommendations about these factors to encourage healthy behaviors. The app includes customizable medication reminders to improve medication adherence [26]. The juli app also encouraged participants to use the positive affect journaling function [27]. The design of the juli app guides participants toward all elements of the app but allows them to flexibly choose where they want to engage.

### Attention-Placebo Control

Participants in the control arm had a limited version of the app. The app prompted participants to open it each day and rate how they were feeling on the 5 emoji scale, but they did not have access to any further functionality or intervention. There was no change to usual care in either arm.

### Assessment Tools

Participants in both arms completed baseline assessments and follow-up assessments at 2, 4, 6, and 8 weeks remotely from within the app. Assessments included the ACT for asthma symptoms and the 12-Item Short Form Health Survey (SF-12) for health-related quality of life. The ACT is a widely used, self-completed asthma symptom scale that is responsive to change with scores ranging from 5 to 25 [28]. A cutoff score of 19 or lower identifies patients with uncontrolled asthma. The SF-12 is a self-reported measure assessing the impact of health on an individual’s everyday life. Scores ranging from 0 to 100 with higher scores indicate a better quality of life [29].

### Outcomes

The total ACT score at 8 weeks was our primary outcome. Secondary outcomes were continuous ACT score at 2, 4, 6, and 8 weeks in a repeated measures analysis using mixed-effect models; remission, defined as a score of >19 at 8 weeks; remission at 2, 4, 6, and 8 weeks in a repeated measures analysis; SF-12 physical and mental component scores at 8 weeks; and SF-12 physical and mental component scores at 4 and 8 weeks in a repeated measures analysis.

We added achieving a minimal clinically important difference (MCID) at 8 weeks (a 3-point increase on the ACT) [30] and a worsening of asthma symptoms (ie, a decrease in ACT scores from baseline) as post hoc outcomes.

### Sample Size Estimation

The best MCID estimate for the ACT is between 2.2 and 3.0 (SD 3.1 to 4.7) [30]. A 2-sided 5% significance level at 80% power requires a total sample size of 146 for an MCID of 3. We aimed to recruit 90 participants per arm, allowing for 23% attrition [31].

### Statistical Analyses

We preprinted the analysis plan on UCL Discovery [32] and preregistered the RCT on the ISRCTN registry with a description of the primary and secondary outcomes before the trial started. In reporting and analyzing our data we followed the CONSORT (Consolidated Standards of Reporting Trials) guidelines [33].

Our primary outcome was the difference in total ACT score at 8 weeks between the control and intervention groups in a per-protocol analysis. We estimated this difference with a linear regression model adjusted for baseline ACT and any imbalanced baseline covariates. We tested how robust the result was to model specification by also using a Poisson model and adjusting for any variables not balanced at baseline. We used logistic regression to calculate the odds ratio (OR) of remission at 8 weeks (ACT>19), achieving MCID (≥3 point ACT improvement), and worsening of asthma, adjusting for baseline ACT. We completed the repeat measures analyses using linear or logistic mixed-effect models adjusting for ACT at baseline.

**Figure 1:**
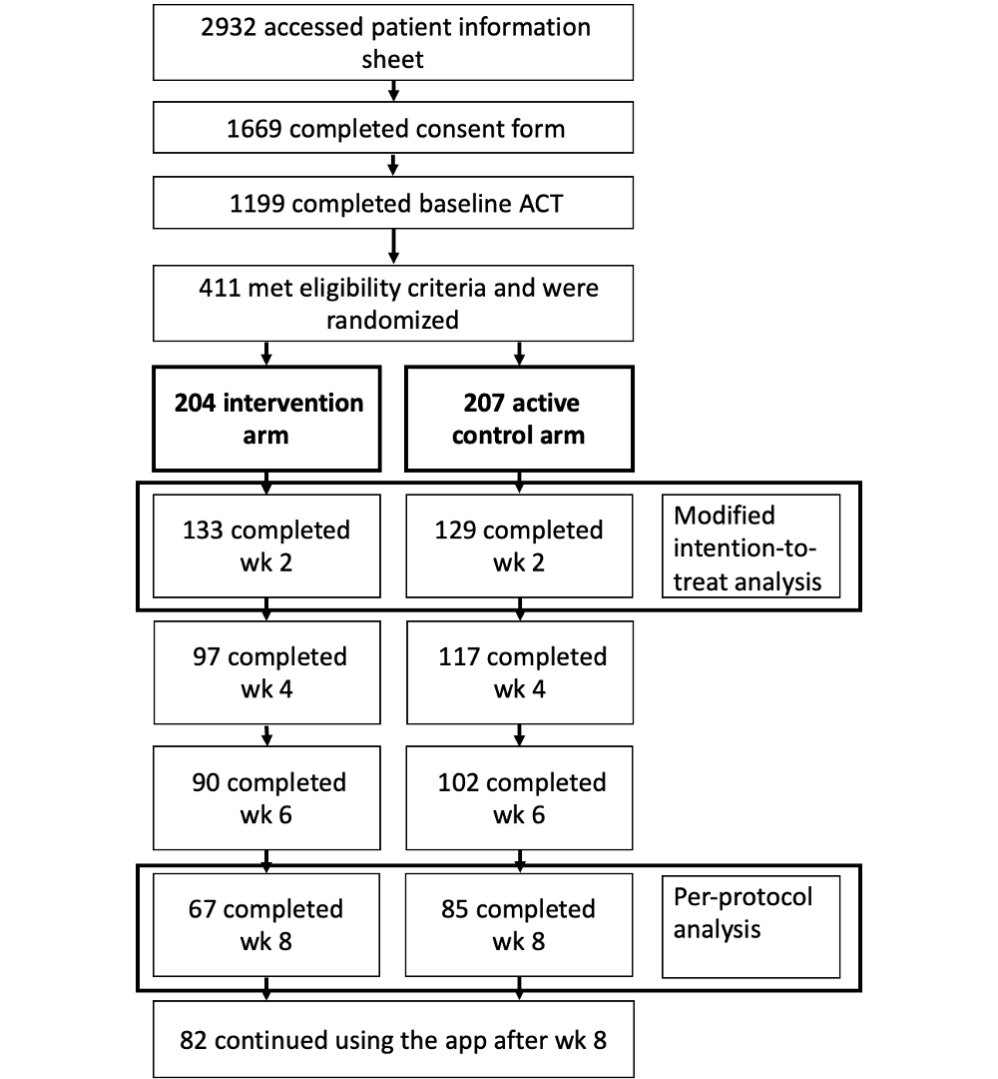
CONSORT diagram. ACT: Asthma Control Test; CONSORT: Consolidated Standards of Reporting Trials.

We repeated the analysis of all outcomes in a modified intention-to-treat (ITT) analysis. This analysis included all randomized participants with a complete baseline and week 2 ACT score, dropping participants who were randomized but never used the app (see Figure 1). We imputed the missing ACT scores first using multiple imputation models and then using the last observation carried forward [34]. The multiple imputation models included predictive mean matching with 5 nearest neighbors and 50 iterations. This method means that only plausible values are imputed and is more robust to model misspecification than fully parametric imputation [35].

An independent statistician with no conflicts of interest with the company providing juli completed the analyses. All analyses we conducted using Stata (version 17; StataCorp) and R (version 4.3.1 for Windows; R Foundation for Statistical Computing).

## Results

### Participants

Of 1199 participants who completed the baseline ACT, 411 (34.3%) participants met eligibility criteria. The 411 participants were randomized: 204 (49.6%) to the intervention arm and 207 (50.4%) to the active control arm. Of the 411 participants randomized, 325 (79.1%) were from the United States. Attrition was similar in both arms: 71 (34.8%) out of 204 participants in the intervention arm and 78 (37.7%) out of 207 participants in the active control arm left this study before the week-2 ACT. The remaining 262 participants contributed to our modified ITT analysis (Figure 1). Further attrition occurred between week 2 and week 8: a total of 66 (49.6%) out of 133 remaining participants left the intervention group, and 44 (34.1%) out of 129 remaining participants left the active control group. The remaining 152 participants contributed to our per-protocol analysis (Figure 1). Participants included in the modified ITT and per-protocol analyses were similar in terms of baseline characteristics (see Table 1 and Multimedia Appendix 1).

**Table 1:** Baseline characteristics of participants completing 8 weeks of the trial, by randomization group. Data used in the per-protocol analysis of individuals completing the week-8 ACT.

Characteristics		Intervention (n=67)	Control (n=85)	All (n=152)
Age (years), mean (SD)		35.73 (11.48)	36.62 (13.23)	36.23 (12.45)
**Sex, n (%)**				
	Female	52 (77.6)	70 (82.4)	122 (80.3)
	Male	13 (19.4)	13 (15.3)	26 (17.1)
	Other	2 (3)	2 (2.4)	4 (2.6)
**Asthma duration, n (%)**				
	<1 month	1 (1.5)	1 (1.2)	2 (1.3)
	1 to <3 months	2 (3)	2 (2.4)	4 (2.6)
	3 months to <1 year	2 (3)	4 (4.7)	6 (4)
	1 year to <2 years	4 (6)	5 (5.9)	9 (5.9)
	2 years to <5 years	11 (16.4)	5 (5.9)	16 (10.5)
	>5 years	47 (70.2)	68 (80)	115 (75.7)
**Physician contact, n (%)**				
	Regular	22 (32.8)	30 (35.3)	52 (34.2)
	Occasional	42 (62.7)	40 (47.1)	82 (54)
	Not anymore	3 (4.5)	10 (11.9)	13 (8.6)
	Never	0 (0)	5 (5.9)	5 (3.3)
**Diagnosed by a physician, n (%)**				
	Yes	67 (100)	81 (95.3)	148 (97.4)
	No	0 (0)	4 (4.7)	4 (2.6)
ACT^a^ total score, mean (SD)		12.60 (4.10)	13.04 (3.93)	12.84 (4.00)
SF-12 physical health subscale^b^, mean (SD)		39.28 (8.95)	39.86 (9.04)	39.61 (8.98)
SF-12 mental health subscale^c^, mean (SD)		38.35 (9.84)	37.74 (10.74)	38.01 (10.32)

^a^ACT: Asthma Control Test (possible range 5-25).

^b^SF-12 physical health subscale: Short-Form Health Survey-12 physical health subscale (possible range 0-100).

^c^SF-12 mental health subscale: Short-Form Health Survey-12 mental health subscale (possible range 0-100).

### Per-Protocol Analysis

Of the 152 participants in the per-protocol analysis, they were mostly female individuals (n=122, 80.3%) who had been diagnosed by a physician more than 5 years ago (n=115, 75.7%) and had ongoing contact with a doctor about their asthma (n=134, 88.2%; Table 1). Participants had a mean baseline ACT score of 12.84 (SD 4.00).

**Figure 2:**
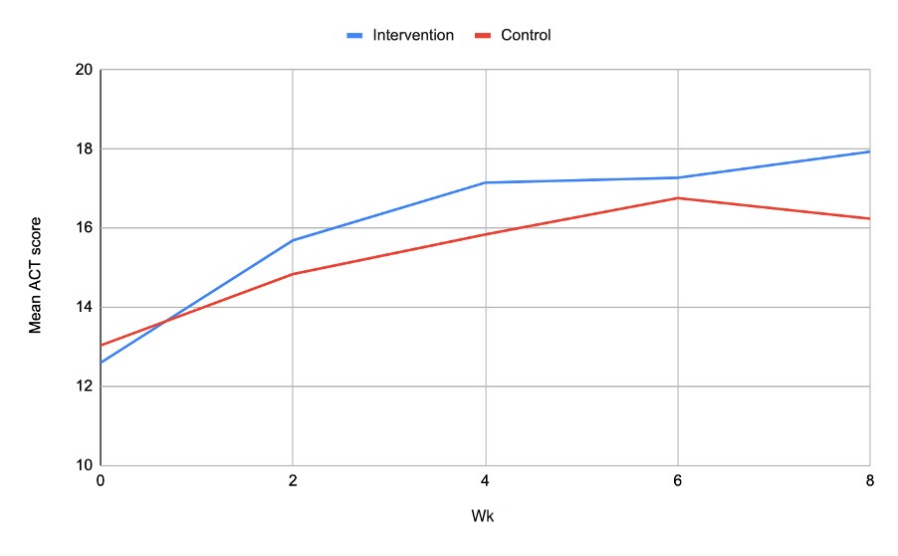
Mean change in ACT score over 8 weeks. ACT: Asthma Control Test.

Intervention group participants had a mean ACT score of 17.93 (SD 4.72) compared with 16.24 (SD 5.78) in the control group after 8 weeks (see Figure 2). After adjusting for baseline ACT score, the intervention group showed a greater improvement in symptom scores at 8 weeks than those in the control group (adjusted coefficient 1.91, 95% CI 0.31-3.51; *P*=.02; Table 2). After adjusting for imbalanced baseline characteristics, the improvement was 2.01 (95% CI 0.48-3.53; *P*=.01) points on the ACT. Using Poisson regression rather than linear regression did not alter our results.

The chance of being in remission by week 8 did not differ between the intervention and control groups after accounting for baseline asthma. However, participants in the intervention group were more likely to experience an MCID (adjusted OR 2.38, 95% CI 1.20-4.70; *P*=.01) than those in the control group. This effect was consistent across the 2-, 4-, 6-, and 8-week assessments (Table 2). The odds of worsening symptoms were similar in both arms (adjusted OR 0.55, 95% CI 0.23-1.32, *P*=.18). There were no between-group differences in SF-12 mental or physical component scores.

**Table 2:** Outcomes of the per-protocol and modified intention-to-treat analyses.

Characteristics		Effect estimate (95% CI)	*P* value
**Per-protocol (n=152)**			
	ACT^a^ at 8 weeks^b^	1.91 (0.31 to 3.51)	.02
	Remission at 8 weeks^c^	1.47 (0.75 to 2.90)	.26
	ACT repeated measures (2, 4, 6, and 8 weeks)^b^	1.34 (0.15 to 2.53)	.03
	Remission repeated measures (2, 4, 6, and 8 weeks)^c^	1.67 (0.62 to 4.52)	.31
	SF-12^d^ physical component score at 8 weeks^b^	0.81 (–1.49 to 3.10)	.49
	SF-12 mental component score at 8 weeks^b^	0.84 (–1.97 to 3.65)	.56
	SF-12 physical component score repeated measures (4 and 8 weeks)^b^	0.611 (–1.32 to 2.54)	.53
	SF-12 mental component score repeated measures (4 and 8 weeks)^b^	0.91 (–1.21 to 3.03)	.40
	MCID^e^ at 8 weeks^c^	2.38 (1.20 to 4.70)	.01
	Worse at 8 weeks^c^	0.55 (0.23 to 1.32)	.18
**ITT^f^, multiple imputation for missing outcomes (n=262)**			
	ACT at 8 weeks^b^	1.56 (0.32 to 2.79)	.01
	Remission at 8 weeks^c^	1.45 (0.80 to 2.63)	.22
	ACT repeated measures (2, 4, 6, and 8 weeks)^b^	1.23 (0.33 to 2.12)	.007
	Remission repeated measures (2, 4, 6, and 8 weeks)^c^	1.40 (0.85 to 2.32)	.19
	SF-12 physical component score at 8 weeks^b^	0.58 (–1.45 to 2.60)	.58
	SF-12 mental component score at 8 weeks^b^	0.73 (–1.75 to 3.22)	.56
	SF-12 physical component score repeated measures (4 and 8 weeks)^b^	0.75 (–1.15 to 2.64)	.44
	SF-12 mental component score repeated measures (4 and 8 weeks)^b^	0.23 (–1.93 to 2.40)	.83
	MCID at 8 weeks^c^	2.17 (1.25 to 3.78)	.006
	Worse at 8 weeks^c^	0.76 (0.39 to 1.56)	.45
**ITT, last observation carried forward for missing outcomes (N=262)**			
	ACT at 8 weeks^b^	1.17 (0.02 to 2.31)	.046
	Remission at 8 weeks^c^	0.92 (0.53 to 1.57)	.75
	ACT repeated measures (2, 4, 6, and 8 weeks)^b^	1.03 (0.12 to 1.93)	.03
	Remission repeated measures (2, 4, 6, and 8 weeks)^c^	0.88 (0.41 to 1.93)	.76
	SF-12 physical component score at 8 weeks^b^	0.17 (–1.67 to 2.02)	.85
	SF-12 mental component score at 8 weeks^b^	0.61 (–1.63 to 2.85)	.59
	SF-12 physical component score repeated measures (4 and 8 weeks)^b^	–0.13 (–2.21 to 1.95)	.90
	SF-12 mental component score repeated measures (4 and 8 weeks)^b^	0.76 (–1.69 to 3.21)	.54
	MCID at 8 weeks^c^	1.95 (1.17 to 3.24)	.01
	Worse at 8 weeks^c^	0.65 (0.35 to 1.21)	.17

^a^ACT: Asthma Control Test.

^b^ coefficient.

^c^Odds ratio.

^d^SF-12: 12-Item Short Form Health Survey.

^e^MCID: minimal clinically important difference.

^f^ITT: intention-to-treat.

### ITT Analysis

The baseline characteristics of participants in the intervention and control groups were similar to the per-protocol analysis. Following multiple imputations of missing outcomes, there was a greater improvement in ACT scores in the intervention group than in the active control group (adjusted coefficient 1.56, 95% CI 0.32-2.79; *P*=.01; Table 2). MCID was more common in the intervention group than the control group (adjusted OR 2.17, 95% CI 1.25-3.78, *P*=.006). Both arms had similar odds of remission, worsening of symptoms, and SF-12 scores. The results from the last observation carried forward analyses were consistent with the per-protocol and multiply imputed results.

## Discussion

### Principal Findings

Our primary analysis showed that juli users had a greater improvement in asthma symptoms at 8 weeks compared to an attention-placebo control. The mean improvement in the intervention group was 5.33 (SD 5.33) compared with 3.20 (SD 5.26) in the control group. This total improvement and the difference between arms are consistent with a clinically important effect of juli on asthma control [30]. Participants assigned to juli had more than twice the odds of a 3-point (MCID) or greater improvement on the ACT. However, the mean ACT score at 8 weeks in both arms fell below the established cut point for “well-controlled” asthma, and there was no difference between arms in terms of odds of remission. The results from our multilevel models covering outcomes from 2 to 8 weeks and the modified ITT analysis with all individuals who were randomized and used the app for at least 2 weeks were consistent with these primary findings.

Participants entering our trial had a mean baseline ACT score of 12.84 (SD 4.00), indicating they fulfilled the GINA definition of “very poorly controlled” (score of 13) as uncontrolled is scores of 19 or lower [20], and most reported having asthma for several years with routine physician contact, suggesting difficulties with long-term asthma control. The results of this trial indicate that juli can augment the treatment of uncontrolled asthma as indicated by improved ACT scores over 8 weeks. There is consistent evidence that low ACT scores are associated with rescue medication use, asthma exacerbations, reduced lung function, and reduced asthma-specific quality of life, sleep, work, and productivity [6]. Increases in ACT scores are associated with decreased health care usage and health care costs [6].

It is unclear which component of juli resulted in improved ACT scores, but participants likely chose elements that suited them, which is a strength of juli’s design, allowing for a degree of self-personalization. Previous research into asthma app functionality has highlighted symptom tracking, clinical questionnaires, goal setting, performance feedback, medication reminders, and tracking as valuable to patients [17]. Gamification and contingent rewards are also important features incorporated into juli [17]. Positive affect journaling is a novel, evidence-based addition to juli’s functionality [36]. Other commercially available apps for adult asthma self-management use similar behavior change techniques, health education, symptom recording, environmental data, medication reminders, and data presentation. A recent review identified over 500 asthma-related mobile and inhaler-based monitoring apps [37]. However, only a small number of these had any degree of scientific evaluation; with positive fully powered trials being rare [37]. An additional problem for patients is the high rate of failure of companies providing these apps, with only a small number with evidence being available currently. These include AsthmaMD (AsthmaMD Inc) [14], Kiss myAsthma (University of Sydney, the Woolcock Institute of Medical Research, and The University of Melbourne) [38], ASTHMAXcel (ASTHMAXcel) [39], and eAMS (EAPOC [Evidence at the Point-of-Care]) [40], each having positive pilot data.

The juli app is available in Android and iOS formats globally. It is a highly accessible platform for people with asthma, and our trial provides methodologically robust evidence of its efficacy in managing asthma. Additional research is required to understand the most cost-effective support procedures to improve adherence to digital self-management tools and how best to integrate them into clinical practice. The majority of the early attrition in our RCT was in participants who never began to use the app. To reduce this, future RCTs of digital interventions may benefit from a run-in period, in which participants become familiarized with the app before randomization [41].

### Strengths and Limitations

There were several strengths and limitations to this RCT. We successfully and remotely recruited, screened, randomized, treated, and assessed participants worldwide. People could easily participate in the trial as our modified version of the juli app allowed consent, randomization, and assessments to occur within the platform. This facilitated a low-cost global recruitment strategy and a pragmatic trial design with good external validity. However, our focus on reducing participant burden limited the types and richness of the data we were able to collect at baseline. For example, we lack relevant information on income, education, and other social determinants of health. Despite this, we did achieve a postrandomization balance in recorded characteristics at baseline, indicating successful randomization. Most of the participants were female, reflecting established differences in sex-specific rates of asthma [42], health behaviors, and health care use in adults [43].

Participants completed the ACT, which is a recommended primary end point in clinical trials for asthma [6]. We also preregistered our primary and secondary outcome measures along with a full analysis plan, which we adhered to. However, we lacked a broader battery of outcome measures that could have further contextualized our findings and identified possible mechanisms of action.

Attrition was greater than we predicted. The attrition in our trial follows a similar pattern to other digital RCTs, including for asthma apps, where it mostly occurs between randomization and week 2. Dropout rates in previous RCTs have ranged from 20% to 60% [10]. However, studies recruiting via social media have had low retention at 30 days (<20%) [44], and a similar, all-remote RCT of mobile health support for asthma had an attrition of 62% at 9 weeks [45]. To manage attrition, we continued recruiting and randomizing participants until we had a sufficient number of participants completing the week 8 outcome measures to meet our sample size calculation. We examined differences in completers versus noncompleters (see Table 1 and Multimedia Appendix 1). There were unlikely to be differences between those who dropped out of this study and those who completed it, based on their baseline characteristics, including asthma severity. Our modified ITT and primary analysis findings were similar, suggesting the intervention would have had a similar effect on those who dropped out. The ITT analysis used 2 imputation methods that make different assumptions [34], and results were consistent using both methods. Despite this, it is impossible to rule out attrition bias and our results should be seen as reflecting the effect on people motivated and able to use juli.

### Conclusions

The juli app has been demonstrated to decrease asthma symptoms within 8 weeks, with an increased chance of achieving MCID, but no difference in terms of odds of remission. As such, juli represents a low-risk and low-cost adjunct to the care regimen of individuals with asthma.

## Appendix

Multimedia Appendix 1 Baseline characteristics of individuals in the modified intention-to-treat analysis.

Multimedia Appendix 2 CONSORT-eHealth checklist (V 1.6.1).

## Abbreviations

ACT: Asthma Control Test
CONSORT: Consolidated Standards of Reporting Trials
GINA: Global Initiative for Asthma
ISRCTN: International Standard Randomised Controlled Trial Number
ITT: intention-to-treat
MCID: minimal clinically important difference
OR: odds ratio
RCT: randomized controlled trial
SF-12: 12-Item Short Form Health Survey

## Data Availability

All data produced in this study are available upon reasonable request to the authors.
