# Supplementary material for: Digital Self-Management Platform for Adult Asthma: Randomized Attention-Placebo Controlled Trial
Source: J Med Internet Res. 2024 Apr 29;26:e50855. doi: 10.2196/50855 (PMC11091812; doi:10.2196/50855)
Supplement: Multimedia Appendix 1 [file jmir_v26i1e50855_app1.docx]

**Multimedia Appendix 1.** Baseline characteristics of individuals in modified intention-to-treat analysis.

|  | Intervention  (n=133) | Control  (n=129) | All  (n=262) |
| --- | --- | --- | --- |
| Age | 34.14 (11.82) | 35.55 (13.54) | 34.84 (12.69) |
| Gender |  |  |  |
| Female | 104 (78.20) | 101 (78.29) | 205 (78.24) |
| Male | 23 (17.29) | 26 (20.16) | 49 (18.70) |
| Other | 6 (4.51) | 2 (1.55) | 8 (3.05) |
| Depression duration |  |  |  |
| <1 month | 2 (1.50) | 1 (0.78) | 3 (1.15) |
| 1 to <3 months | 4 (3.01) | 4 (3.10) | 8 (3.05) |
| 3 months to <1 year | 9 (6.77) | 8 (6.20) | 17 (6.49) |
| 1 year to <2 years | 9 (6.77) | 7 (5.43) | 16 (6.11) |
| 2 years to <5 years | 20 (15.04) | 11 (8.53) | 31 (11.83) |
| >5 years | 89 (66.92) | 98 (75.97) | 187 (71.37) |
|  |  |  |  |
| Physician contact |  |  |  |
| Regular | 43.(32.33) | 41 (31.78) | 84 (32.06) |
| Occasional | 76 (57.14) | 70 (54.26) | 146 (55.73) |
| Not anymore | 12 (9.02) | 12 (9.30) | 24 (9.16) |
| Never | 2 (1.50) | 6 (4.65) | 8 (3.05) |
| Diagnosed by a physician |  |  |  |
| Yes | 131 (98.50) | 124 (96.12) | 255 (97.33) |
| No | 2 (1.50) | 5 (3.88) | 7 (2.67) |
| ACT total score^1^ | 13.01 (4.24) | 12.97 (4.42) | 12.99 (4.32) |
| SF-12 physical health subscale^2^ | 39.94 (8.85) | 40.02 (8.53) | 39.98 (8.67) |
| SF-12 mental health subscale^3^ | 38.29 (9.49) | 38.50 (10.53) | 38.39 (9.99) |

Data are n (%) or mean (SD). ^1^Patient Health Questionnaire, 8-item version (possible range 0–24), ^2^Short-Form Health Survey-12 physical health subscale (possible range 0–100), ^3^Short-Form Health Survey-12 mental health subscale (possible range 0–100).
